# Computational Phosphosite-Specific Network Analysis of YES1 Y426 Reveals Cancer-Associated Phosphorylation Patterns

**DOI:** 10.3390/proteomes14020017

**Published:** 2026-04-16

**Authors:** Afreen Khanum, Leona Dcunha, Suhail Subair, Athira Perunelly Gopalakrishnan, Akhina Palollathil, Rajesh Raju

**Affiliations:** Centre for Integrative Omics Data Science (CIODS), Yenepoya (Deemed to be University), Mangalore 575018, Karnataka, India; afreenkhan5031@gmail.com (A.K.); leonadcunha@gmail.com (L.D.); suhailsubair.ciods@yenepoya.edu.in (S.S.); 19228@yenepoya.edu.in (A.P.G.)

**Keywords:** phosphoproteomics, predominant phosphosite, cancer biomarkers, phosphoregulatory networks

## Abstract

Background: YES1 is an Src family non-receptor tyrosine-protein kinase that regulates cell growth, migration, survival, and oncogenic signaling. Although YES1 activation mechanisms and substrates have been extensively studied, its phosphosite-specific regulation across diverse biological contexts remains poorly understood. Methods: We performed a large-scale integrative analysis of 3825 publicly available human mass spectrometry-based phosphoproteomic datasets to map YES1 phosphorylation events. Co-modulation, co-occurrence, evolutionary conservation, and disease-association analyses were conducted to characterize the functional and clinical relevance of site-specific YES1 phosphorylation. Results: Y426 emerged as the predominant YES1 phosphosite across diverse biological conditions, localized within the activation loop of the kinase domain and conserved across Src family kinases. Co-modulation analysis identified 421 positively and 102 negatively associated phosphosites enriched in biological processes related to cell cycle regulation, transcription, cytoskeletal remodeling, apoptosis, and carcinogenesis. Among these high-confidence protein phosphosites, we identified 24 binary interactors, 5 upstream regulators, and 8 candidate downstream substrates. Comparison with DisGeNet cancer biomarkers showed overlap between YES1-associated phosphoproteomic signatures and site-specific oncogenic markers across multiple cancers, such as breast cancer, colorectal cancer, leukemia, and lung adenocarcinoma. Conclusions: This study provides a systems-level, phosphosite-focused view of YES1 signaling and supports a central regulatory role for Y426 within global phosphoregulatory and cancer-associated networks.

## 1. Introduction

YES1 (tyrosine-protein kinase Yes), also called YES proto-oncogene 1, is a non-receptor tyrosine kinase encoded by the *YES1* gene located on chromosome 18p11.32 in humans. It is the cellular homolog of the Yamaguchi sarcoma virus oncogene, marking its initial discovery in studies of viral oncogenes; the human ortholog was subsequently cloned and characterized in the mid-1980s [1,2]. The human YES1 protein is composed of 543 amino acids [3]. Subcellular localization studies reveal that YES1 is predominantly found in the cytoplasm; however, it is also associated with cellular membranes, such as the plasma membrane, and can undergo nucleocytoplasmic shuttling, depending on cellular context and signaling states [4].

YES1 regulates diverse cellular processes such as cell adhesion, cell growth, survival, cytoskeleton organization, apoptosis and cell differentiation [5]. YES1 transduces signals from multiple receptor tyrosine kinases, playing pivotal roles in mediating phosphorylation cascades that control cell proliferation and motility, a function directly implicated in oncogenic processes and disease states [6,7]. A key indicator of YES1 kinase activation and its downstream signaling is the phosphorylation of Y426 by upstream receptor tyrosine kinases, G-protein-coupled receptors, or integrins [8]. Y426 is also a known autophosphorylation site of YES1 kinase [9]. YES1 participates in multiple signaling pathways involving Wnt/β-catenin, PI3K/AKT, MAPK/ERK, and Hippo signaling. Evidence from previous studies suggests that YES1 promotes cancer cell migration and invasion in a PI3K/AKT signaling-dependent manner [10,11]. YES1-mediated phosphorylation of YAP1 (Yes-Associated Protein 1), a downstream effector in Hippo signaling, modulates transcriptional programs promoting oncogenesis [12]. YES1 also influences tight junction assembly by phosphorylating junctional proteins, such as Partitioning Defective 3 (PARD3), regulating epithelial barrier integrity [4,7]. It also regulates cell adhesion through interaction with actins, integrins, and focal adhesion kinases [13]. Dysregulation of YES1 is implicated in oncogenic transformation and progression in numerous cancers, including lung, breast, ovarian, colorectal, and skin cancers [14,15,16,17,18]. YES1 amplification, overexpression, or aberrant activity correlates with enhanced tumor growth, metastasis, and poor patient prognosis [16,19]. YES1 also contributes to resistance to targeted cancer therapies such as EGFR and Anaplastic Lymphoma Kinase (ALK) inhibitors, underscoring its clinical relevance as a potential therapeutic target [5,20]. Dasatinib is a short-acting dual ABL/SRC tyrosine kinase inhibitor used to treat certain leukemias. It inhibits BCR-ABL and members of the SRC family kinases, including SRC, LCK, YES1, and FYN, and also targets KIT, PDGFRA, PDGFRB, and members of the Eph receptor family [5,20]. Dasatinib effectively inhibits YES1 kinase activity and reverses resistance to EGFR and HER2 inhibitors in cancer models [21]. Emerging selective YES1 inhibitors (e.g., CH6953755) show promise for improved therapeutic specificity. Combination therapies targeting YES1 alongside primary oncogenic drivers are being explored to overcome drug resistance and improve treatment outcomes [5,22].

Although YES1 is a well-established oncogenic Src family kinase, a global and systematic understanding of the co-occurrence, coordination, and network-level modulation of its phosphorylation events remains limited. Given the central role of the activation-loop phosphosite Y426 in YES1 kinase activity, we focused our analysis on this predominant phosphosite, examining its co-occurrence patterns and identifying protein phosphosites that exhibit significant co-modulation with Y426 across large-scale human phosphoproteomic datasets. This integrative analysis aims to define coordinated phosphorylation modules and elucidate how YES1-centered signaling networks contribute to key biological processes and oncogenic pathways.

## 2. Materials and Methods

### 2.1. Systematic Screening and Integration of YES1 Phosphoproteomic Data to Identify Predominant YES1 Phosphosites

To screen and assemble global phosphoproteomic datasets, we conducted a PubMed search using the query term “phosphoproteome” OR “phosphoproteome” NOT “plant” NOT “review”. We systematically screened the available published human mass spectrometry-based global phosphoproteome datasets to retrieve Class-1 phosphosites in YES1 (localization probability greater than or equal to 75% or A-score greater than or equal to 13). These datasets were generated from the LC-MS/MS analysis of LysC and/trypsin-digested peptides. The dataset was divided into two categories: a quantitative differential dataset, where biological or experimental conditions (tests) are compared against corresponding controls, and a profile dataset, where tests and controls were analyzed independently. Additionally, the datasets were grouped according to a phosphosite enrichment approach (STY/ST/Y phosphosites). In the quantitative differential profile, Class-1 phosphosites showing ≥1.3 or ≤0.76-fold change were defined as increased or decreased according to study-centric statistical thresholds. Proteins with a *p*-value less than 0.05 were considered significant. These thresholds were applied to improve the data’s biological relevance and maintain statistical significance. Each protein was mapped to its related gene symbols in accordance with the most recent guidelines from the HUGO Gene Nomenclature Committee (HGNC) [23]. Furthermore, each phosphoprotein within the datasets was linked to its corresponding UniProt (13.04.2023) [24] accession using a self-developed mapping tool to maintain consistency. Annotated biological and experimental conditions were assigned to each dataset in a standardized format to facilitate effective categorization [25].

**Figure 1:**
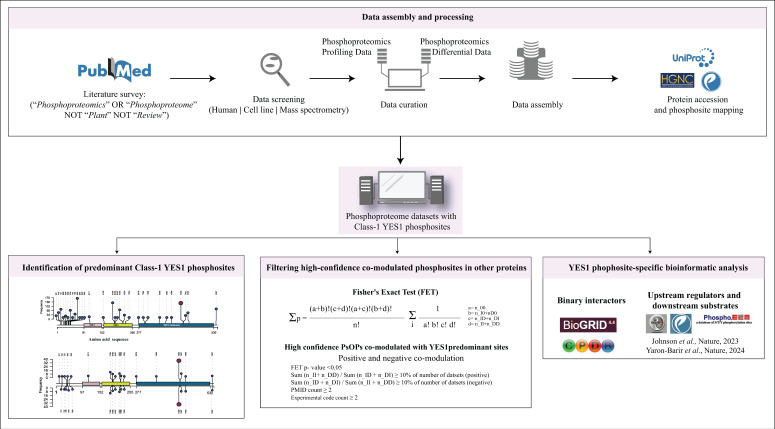
Workflow for screening, curation and assembling of phosphoproteomic datasets and analyzing YES1 phosphosites along with their co-modulated phosphosites in other proteins. Human cell line mass spectrometry data were compiled and mapped to the latest UniProt release. The predominant YES1 phosphosite Y426 was identified from qualitative profiling and quantitative differential datasets and assessed for bias. Pairwise co-modulations within YES1 phosphosites and between YES1 and other proteins were evaluated through one-sided Fisher’s exact tests. High-confidence co-modulated phosphosites (positive/negative) were filtered using strict thresholds, followed by enrichment analysis among known/predicted YES1 interactors, upstream regulators, candidate downstream regulators and kinase/phosphatase networks to infer functional roles of key YES1 phosphosite Y426 [41,42].

Class-1 YES1 phosphosites were retrieved from the compiled mass spectrometric-based human cellular phosphoproteome profile datasets. The phosphosites of YES1 were computed, scored and ranked based on the frequency of their detection in qualitative profile datasets and quantitative datasets. Phosphosites that appear frequently in both the overall qualitative profile datasets and the quantitative differential datasets were identified as predominant phosphosites of the YES1 kinase, while phosphosites identified using specific phospho-antibodies or mutation-based approaches were excluded if they were not frequently reported as Class-1 sites in phosphoproteomic datasets due to dataset limitations. The analysis followed a specific methodology, which is outlined in Figure 1.

### 2.2. Co-Occurrence Analysis of YES1 Phosphosites

To examine the mutual associations among phosphosites within YES1, we conducted a co-occurrence analysis focused on the co-modulation patterns of phosphosite pairs. Each differential dataset with various phosphosites of YES1 discovered under the same experimental condition was selected. For individual phosphosite pairs, the frequency of II (increased abundance of both YES1-Y426 and PsOP), ID (YES1-Y426 with increased abundance, while PsOP with decreased abundance), DI (YES1-Y426 with decreased abundance, while PsOP with increased abundance), and DD (both YES1-Y426 and PsOP with decreased abundance) was separately calculated, and their positive co-modulation pattern using ∑ (nII + nDD)/∑ (nID + nDI) and negative co-modulation pattern using ∑ (nID + nDI)/∑ (nII + nDD) were further assessed. A similar approach was used in our laboratory for previous work [26]. The phosphosite pair differential co-modulation frequency is listed in Supplementary Table S1.

### 2.3. Detection of Protein Phosphosites Co-Modulated with the Predominant YES1 Phosphosite

Quantitative differential datasets for predominant phosphosite Y426 were categorized to detect the phosphosites in other proteins (PsOPs) which exhibit positive or negative abundance with YES1. Due to heterogeneity in the experimental conditions, biological systems, and distinct analytical platforms among these datasets, it was not possible to reexamine the raw datasets However, to ensure reliability, only phosphosites with a *p*-value < 0.05, localization probability greater than or equal to 75% and an A-score greater than or equal to 13 were considered for further analysis [27,28].

The differential abundance datasets reporting YES1-Y426 phosphosites were grouped into (i) datasets with increased abundance (It) of pY426 and (ii) datasets with decreased abundance (Dt) of pY426. The PsOPs either increased (Io) or decreased (Do) in relation to the predominant site (Y426) in each category were extracted, leading to four groups: ItIo (both YES1-Y426 and PsOP increased), ItDo (YES1-Y426 increased, while PsOP decreased), DtIo (YES1-Y426 decreased, while PsOP increased), and DtDo (both YES1-Y426 and PsOP decreased). Subsequently, the frequency of datasets exhibiting each of these modulatory relationships with YES1-pY426 was computed. PsOPs in the ItIo and DtDo categories were presumed to be positively correlated with YES1-pY426 abundance, while those in ItDo and DtIo were assumed to be negatively correlated. Accordingly, PsOPs were further grouped into two categories: ItIoDtDo (referred to as positive co-modulation with YES1 phosphosite) and ItDoDtIo (referred to as negative co-modulation with YES1 phosphosite).

### 2.4. Filtering Phosphosites to Minimize Bias and Redundancy

The likelihood and confidence of phosphosite co-modulation were assessed by constructing contingency tables followed by one-sided Fisher’s exact testing (FET). The positive (IIDD) and negative (IDDI) co-modulation patterns with a FET *p*-value threshold of <0.05 were considered statistically significant. High-confidence co-modulation patterns among significantly co-modulated PsOPs were subsequently determined by calculating the co-modulation ratio. For positive co-modulation, the ratio was defined as ∑(nItIo + nDtDo)/∑(nItDo + nDtIo), whereas for negative co-modulation, it was calculated as ∑(nItDo + nDtIo)/∑(nItIo + nDtDo). Similar approaches have previously been employed to investigate co-occurring phosphosites [29]. Phosphosites were designated as high-confidence when the ratio exceeded 10% of the total number of differential datasets for the YES1 phosphosite. For instance, a ratio cutoff of 7.2 was applied for PsOPs co-modulated with YES1–pY426, since YES1–pY426 was differentially modulated in 72 quantitative datasets.

Phosphosite filtration was conducted to prevent biases and over-representation in datasets that arise from having multiple time points within an individual study or similar experimental conditions across different research. Filtering was applied to account by requiring a minimum of three distinct studies (PubMed IDs) and three unique experimental conditions. High-confidence PsOPs meeting all these criteria were used for further analyses of signaling pathways, biological processes, interactions, and kinase–substrate relationships.

### 2.5. Mapping of Protein Interaction Partners Associated with YES1

The experimentally identified protein–protein interactors, including their substrate, were extracted from different databases, such as BIND (Biomolecular Interaction Network Database, Blueprint Initiative, Toronto, Ontario, Canada) [30], HPRD (Human Protein Reference Database, Institute of Bioinformatics, Bangalore, India) [31], ConsensusPathDB version 35 (Berlin, Germany) (downloaded on 22.05.2023) [32], BioGRID (The Biological General Repository for Interaction Datasets, Toronto, Ontario, Canada) [33], RegPhos 2.0 (Taiwan, China), (downloaded on 24.05.2023) [34] and CORUM (The comprehensive resource of mammalian protein complexes, Neuherberg) (downloaded on 03.03.2023) [35].

### 2.6. Retrieval of Potential Upstream Regulators and Candidate Downstream Substrates of YES1

Experimentally validated upstream regulators associated with YES1 phosphosites, as well as YES1 substrates along with their specific phosphosites, were retrieved from multiple databases, including Phospho.ELM 9.0 (European Bioinformatics Institute (EMBL-EBI), Hinxton, Cambridge, United Kingdom) (downloaded on 24 May 2023) [36], RegPhos 2.0 (Taiwan, China), (downloaded on 24 May 2023) [34] and PhosphositePlus (Cell Signaling Technology, Danvers, MA, USA), (downloaded on 22 May 2023) [37]. Additionally, predicted kinases for specific YES1 phosphosites and YES1 substrates were identified using multiple computational tools, such as AKID (Automatic Kinase-specific Interactions Detection) (downloaded on 24 May 2023) [38] and NetworKIN (Samuel Lunenfeld Research Institute, Mount Sinai Hospital, Toronto, Canada) (downloaded on 4 January 2023) [39]. Further, kinase–substrate relationships were derived from a high-throughput in vitro screening of kinases available in iKiP-DB (Berlin) [40]. Since all of these tools required each protein sequence as input, RefSeq protein sequences were retrieved across these tools through the API. Additionally, all YES1 substrates and kinases identified by Johnson et al.(2023) [41,42] through synthetic peptide screening for kinase substrate specificity assessment, with a 90 percentile cutoff, were retrieved for further analysis.

### 2.7. Visualization of the Data

The representation of data was carried out using multiple tools and software. The trackViewer package from R/Bioconductor 10.18129/B9.bioc.trackViewer was used for generating lollipop plots. The phosphosites cumulative distribution within quantitative differential datasets was depicted using Pandas (version 3.0.1) and Matplotlib (version 3.10.8) in Python. MEGA12 (Molecular Evolutionary Genetic Analysis Version 12) [43] was used for phylogenetic analysis of SRC family kinases. RAWGraphs 2.0 was employed to create dendrograms representing the biological processes of proteins of other phosphosites that co-modulate with our predominant YES1 phosphosite.

## 3. Results

### 3.1. Analysis of Global Human Cellular Phosphoproteomic Datasets Identifies Y426 as Predominant YES1 Phosphosite

**Figure 2:**
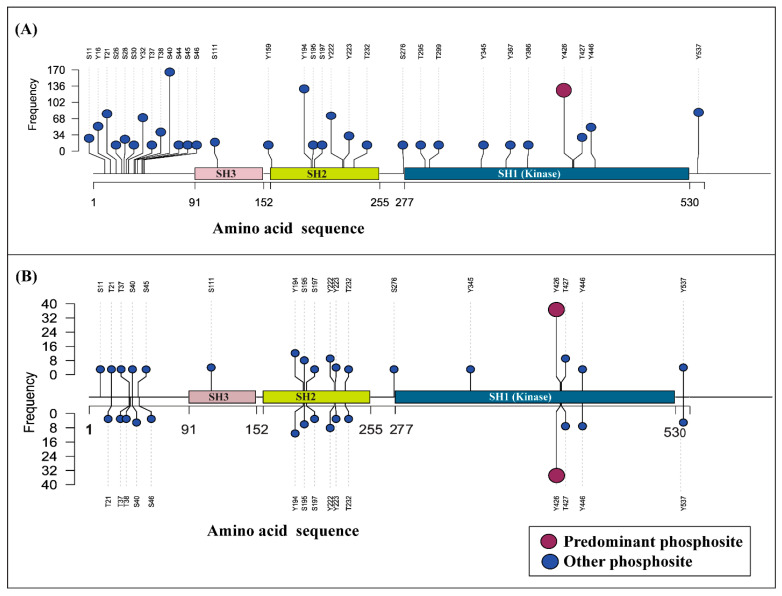
The frequency of YES1 phosphosite identification in qualitative profiling and quantitative differential phosphoproteome studies. Each node in this plot represents the reported phosphosites. (**A**) Frequency of predominant phosphosites identified in the quantitative phosphoproteome profiling studies and (**B**) studies showing quantitative differential abundance of phosphopeptides of YES1. The *X*-axis represents the phosphosite in the YES1 amino acid sequence, while the *Y*-axis represents the frequency of YES1 phosphosites identified from the profiling and differential datasets.

Significant progress has been made in characterizing YES1 activation, phosphorylation sites, interacting partners, upstream regulators, and candidate downstream substrates. However, these insights were largely studied across individual experimental systems, making it difficult to determine the global relevance, recurrence, and coordination of YES1 phosphorylation events. Consequently, a systems-level understanding of how individual YES1 phosphosites are recurrently modulated across diverse biological contexts, and how these sites integrate into broader phosphoregulatory networks, remains limited. We analyzed over 3825 publicly available human global cellular mass spectrometric-based phosphoproteomics datasets and identified 440 qualitative profiling datasets and 133 quantitative differential datasets with Class-1 YES1 phosphosites (Supplementary Tables S2 and S3). Systematic mapping revealed 31 YES1 phosphosites from the qualitative profiling datasets and 20 YES1 phosphosites from quantitative differential datasets (Figure 2). A comprehensive differential abundance profile of YES1 phosphosites was also compiled across diverse experimental conditions. Profiling datasets revealed that S40, Y194, and Y426 are the top frequency phosphosites in 210, 159, and 142 datasets. Among differential phosphoproteome datasets, phosphosite Y426 was detected in 72 datasets. As phosphosite Y426 is frequently reported in both profiling and differential datasets, it was considered a predominant phosphosite. This predominant phosphosite is situated within the activation loop of the kinase domain (PaKD) [44], and phosphorylation at Y426 serves as the key event that stimulates the catalytic activity of YES1 [8].

### 3.2. Structural Features and Evolutionary Conservation of YES1

YES1 kinase belongs to the Src family of tyrosine kinases, a well-conserved group that includes SRC, FYN, LYN, LCK, HCK, BLK, and FGR as core members [20]. comprising three domain regions, namely, SH1, SH2, and SH3, alongside a short motif called SH4 and a unique region. These regions are conserved among the family members, except for the unique domain region. The SH1 domain, also called the tyrosine kinase domain, serves as the catalytic core that mediates a protein’s tyrosine kinase activity, which involves the phosphorylation of substrate proteins. This domain plays a critical role in YES1’s involvement in various cellular processes, including cell growth, survival, differentiation, and migration [45]. The SH2 domain recognizes specific tyrosine phosphosites via a conserved binding pocket containing arginine. It also possesses a binding pocket that recognizes hydrophobic residues positioned C-terminally to the phosphotyrosine motif [45,46]. The SH3 domain, consisting of 60 amino acid residues, mediates protein–protein interactions by targeting proline-rich sequences [8,47]. The SH4 motif is located at the N-terminal region, where it directs membrane localization and biological activity through lipid modifications such as myristylation and palmitylation. The motif constitutes a signature sequence (Met-Gly-X-X-X-Ser/Thr) that serves as the site for myristate attachment. This modification irreversibly anchors YES1 to the membrane and is fundamental for its role in cellular signaling and transformation [48]. The unique domain, a poorly conserved, disordered, and highly variable region situated between the SH4 and SH3 domains, is the primary structural feature that differentiates the Yes1 protein from other Src family members, conferring unique regulatory functions and determining specific protein–protein interactions [49].

**Figure 3:**
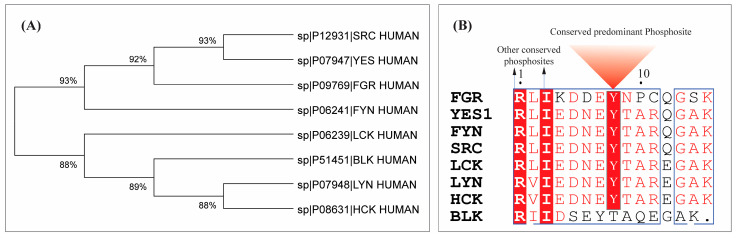
(**A**) Phylogenetic analysis of human SRC family kinases. (**B**) Conservation of predominant phosphosite across SRC family kinases.

In non-receptor tyrosine kinases, the arrangement of the SH3–SH2 kinase domain has been conserved throughout evolution, maintaining an identical arrangement from unicellular choanoflagellates, the earliest organisms possessing Src family kinases, to higher animals. The spatial arrangement of these domains is essential for enzyme regulation [50,51,52]. Multiple sequence alignment using the T-Coffee Expresso tool reveals an alignment score of 99, and phylogenetic analysis conducted using Mega12 (Figure 3A), underscoring their close evolutionary relationship. Site-specific conservation reveals that the predominant site Y426 of YES1 is also conserved in FGR (Y412), FYN (Y420), SRC (Y419), LCK (Y394), LYN (Y397), and HCK (Y411), as presented in Figure 3B, and all these phosphosites are located within the activation loop of the kinase domain. As reported in the Phosphosite Plus database, phosphorylation of SRC, LCK, and LYN at the phosphosites Y419, Y394, and Y397 is known to induce their enzymatic activity.

### 3.3. Positional Mapping and Co-Occurrence of YES1 Phosphosites

**Figure 4:**
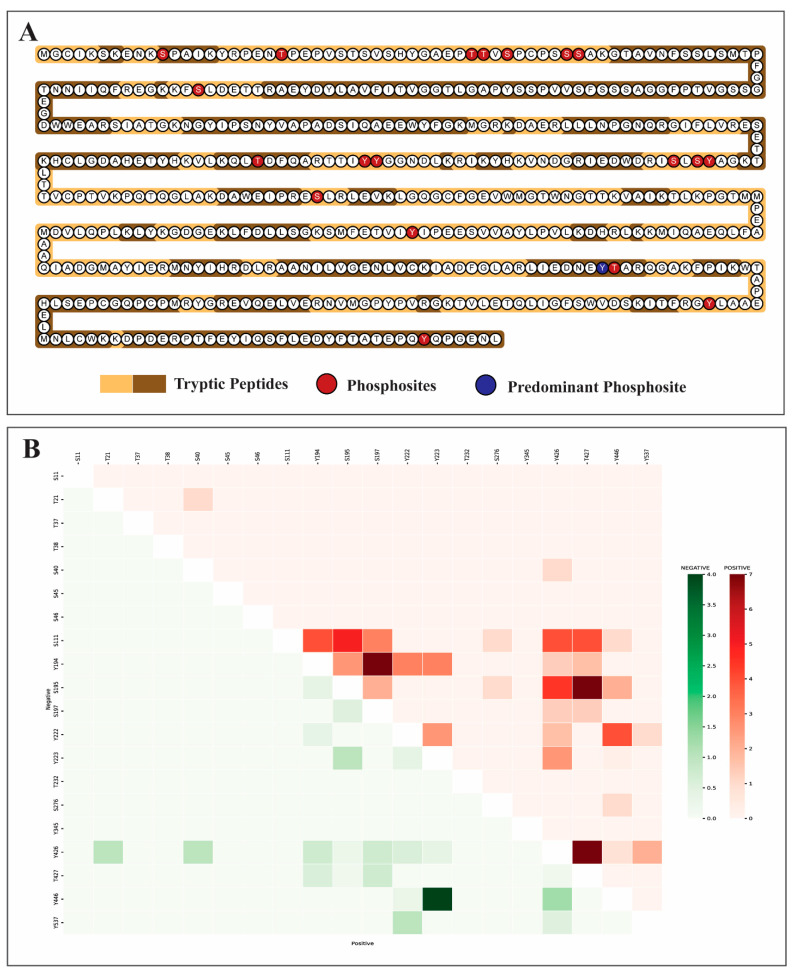
Phosphosites in YES1 and their co-occurrence patterns. (**A**) Visualisation of tryptic peptides within YES1 generated using the ExPASyPeptideCutter tool. Each circle represents individual amino acid residues within the protein sequence. Phosphosites in YES1 highlighted in red and the predominant phosphosite highlighted in blue. (**B**) Heatmap illustrating the co-occurrence patterns of YES1-associated phosphosites. The upper red triangle denotes positive abundance co-modulation, while the lower green triangle represents negative abundance co-modulation between YES1 phosphosite pairs. The color gradient reflects the frequency of these co-modulatory events.

To determine the positional localization of YES1 phosphosites within peptides, tryptic peptides were generated using the ExPASyPeptideCutter tool (Figure 4A). A co-occurrence analysis was then performed to identify the relationship among the YES1 phosphosites. The co-occurrence matrix revealed that predominant phosphosite Y426 is strongly co-occurring with S111, S195 and T427 phosphosites (Figure 4B). Beyond Y426, S111 shows co-occurrence with Y194, S195, and T427, while S195 also co-occurs with T427. Additionally, Y194 shows co-occurrence with S197.

### 3.4. Co-Modulated Protein Phosphosites Associated with the Predominant YES1 Phosphosite Y426

**Figure 5:**
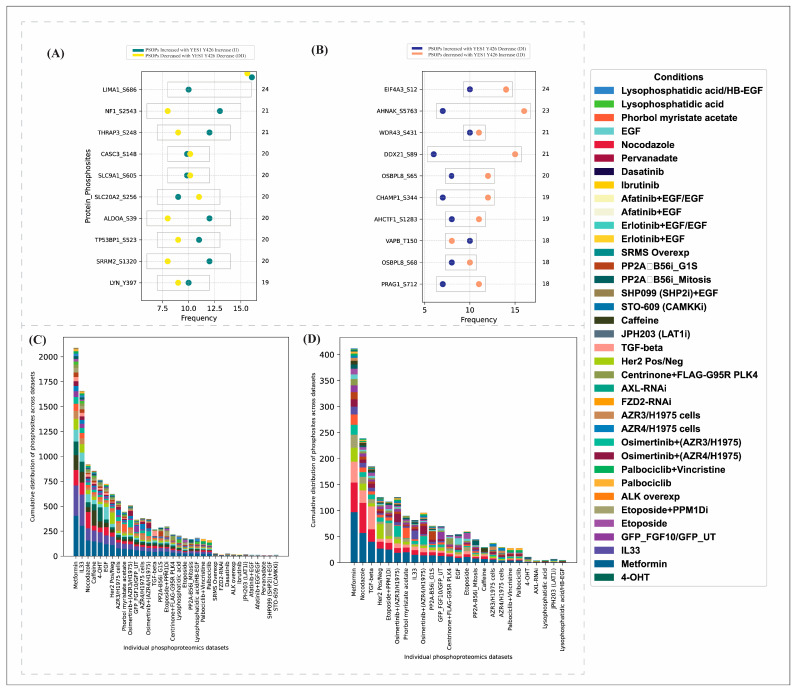
(**A**) Frequency of top 10 positively correlated PsOPs with YES1 (Y426) in datasets where both are increased or decreased. (**B**) Frequency of top 10 negatively correlated PsOPs with YES1 (Y426) in datasets where one is increased, and the other is decreased. (**C**) Cumulative distribution of PsOPs (FET *p* < 0.05) positively modulated with predominant YES1 phosphosite Y426. (**D**) Cumulative distribution of PsOPs (FET *p* < 0.05) negatively modulated with predominant YES1 phosphosite Y426.

Our analysis identified 421 high-confidence PsOPs that were positively co-modulated with Y426, whereas 102 PsOPs showed negative co-modulation, as presented in Supplementary Tables S4 and S5. The high-confidence top 10 protein phosphosites that are positively and negatively co-modulated with predominant phosphosites of YES1 are presented in Figure 5A,B. The cumulative distribution of differential PsOPs with the predominant site of YES1 across different experimental codes is visualized (Figure 5C,D). The top experimental codes, such as metformin, IL-33, caffeine, EGF, and HER2, associated with colorectal cancer, leukemia, hepatocellular carcinoma, lung cancer, and breast cancers, respectively, revealed 9 PsOPs that are common among all these experimental conditions; 56 common PsOPs shared between metformin, IL-33 and caffeine; 27 common PsOPs in metformin, IL-33, caffeine and EGF; and 45 common PsOPs shared among metformin, IL-33 and HER2 (Supplementary Figure S1). This shows that even though these PsOPs are co-modulated across different experimental codes, there is minimal bias towards a particular code and the PsOPs are distributed across these codes.

**Figure 6:**
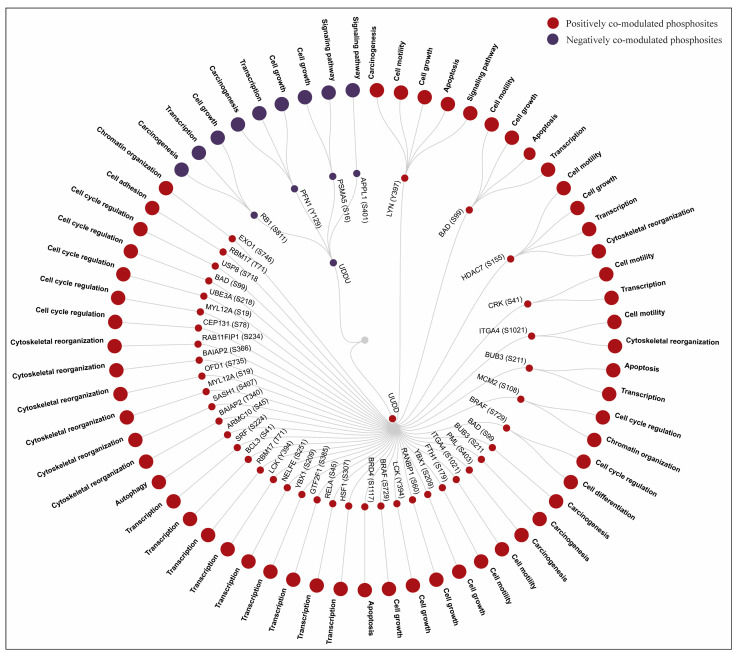
Biological processes of individual protein phosphosites that are co-modulated with YES1 phosphosite Y426.

Since SRC Y419 and LYN Y397, corresponding to the YES1 Y426 conserved activation loop site, are known to play a role in cell cycle progression, apoptosis, and cytoskeletal dynamics [53,54,55], we further analyzed our data to examine whether YES1 may also have similar roles. From our data, we identified 11 PsOPs involved in cell cycle regulation, 9 in cytoskeletal organization, and 5 in apoptosis-related processes. These findings suggest that the phosphorylation of YES1 at Y426 may be associated with regulatory networks involved in cell cycle progression, cytoskeletal organization, and apoptosis. From the analysis of the co-modulated datasets, we identified several biological processes involving positively and negatively co-modulated PsOPs, in which YES1 also participates in similar biological functions (Figure 6), such as cell growth, differentiation, cell motility, apoptosis, transcription, cell adhesion, chromatin organization, signaling pathway regulation, autophagy, cytoskeletal reorganization, carcinogenesis, and cell cycle regulation (Supplementary Table S6).

For example, among positively co-modulated PsOPs, BUB3 (S211) is reported to be linked to transcription [56], and SAM and SASH1 (S407) to cytoskeletal reorganization [57]. LYN (Y397) and BAD (S99) are involved in the regulation of cell motility and apoptosis, as well as in carcinogenesis [58,59,60]. YBX1 (S209) is involved in cell growth [61], and CEP131 (S78) and UBE3A (S218) in cell cycle regulation [62,63]. LYN (Y397) and SASH1 (S407) are involved in the signaling pathway. MCM2 (S108) is involved in chromatin organization and carcinogenesis [64,65]. In negatively co-modulated PsOPs, RB1 (S811) and PFN1 (Y129) [66,67] are involved in transcription and carcinogenesis. Additional functions among positively co-modulated PsOPs, where YES1 is not directly implicated, include RNA stability (ZFP36 (S60)), exocytosis (MYL12A (S19)), and RNA splicing (RBM17 (T71), BRAF (S729), and ITGA4 (S1021)).

### 3.5. Site-Specific Phosphoregulation of YES1 Binary Interactors

**Figure 7:**
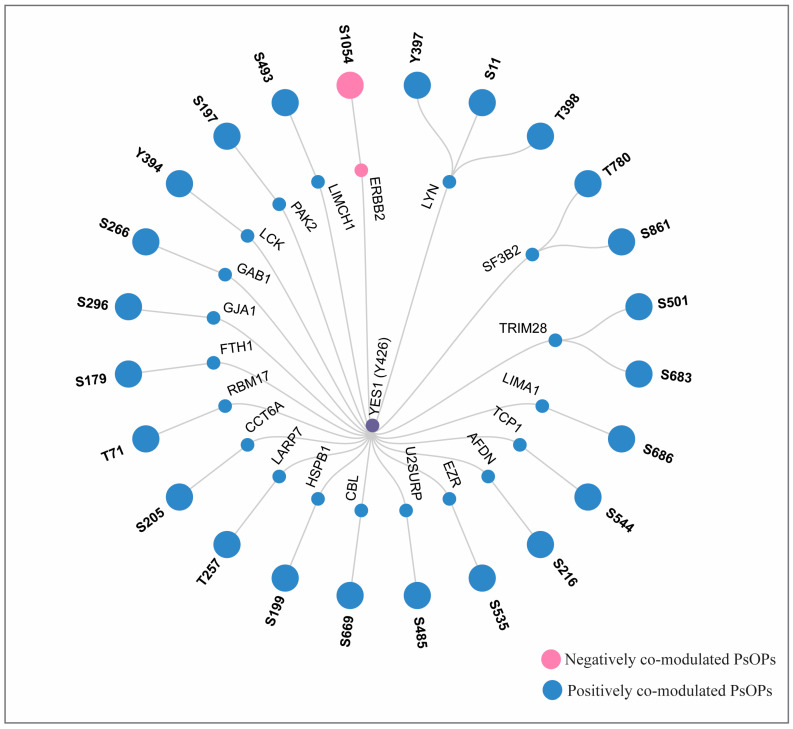
Binary interactors of YES1 phosphosite Y426. Purple color represents YES1 phosphosite Y426, blue color represents binary interactors that are positively co-modulated with YES1 phosphosite Y426, and pink color represents binary interactors that are negatively co-modulated with YES1 phosphosite Y426.

To identify the site-specific phosphorylation dynamics of YES1, the interacting partners were compiled from various resources, including BIND, HPRD, ConsensusPathDB version 35, BioGRID, RegPhos 2.0 and CORUM [30,32,33,34,35,68]. The compiled data included 24 binary interactors of YES1. Of these, positive co-modulation (IIDD) accounted for 23 binary interactors, whereas negative co-modulation (IDDI) comprised 1 binary interactor (Supplementary Table S7). Specifically, LIMA1 (S686), LYN (Y397), TCP1 (S544), AFDN (S216), SF3B2 (T780), EZR (S535), TRIM28 (S501), U2SURP (S485), CBL (S669), LYN (S11), TRIM28 (S683), HSPB1 (S199), LARP7 (T257), CCT6A (S205), RBM17 (T71), FTH1 (S179), GJA1 (S296), SF3B2 (S861), GAB1 (S266), LCK (Y394), LYN (T398), PAK2 (S197), and LIMCH1 (S493) were the top positively co-modulated binary interactors and ERBB2 (S1054) was the negatively co-modulated binary interactor. Interestingly, some of the positively co-modulated binary interactors displayed more than one modulated phosphosite, such as LYN (S11, T398, and Y397), SF3B2 (S861 and T780), and TRIM28 (S501 and S683), indicating coordinated modulation at the phosphosite level (Figure 7).

### 3.6. Potential Upstream Regulators and Candidate Downstream Substrates Co-Modulated with YES1 Y426

Numerous signaling modules exist concurrently and interact significantly under different biological circumstances; however, these modules have not yet been represented on a large scale. Analysis of global phosphoproteome datasets tends to focus mainly on kinases, substrates, and interactors. The global phosphoproteome datasets compiled in our present study provide a suitable platform to derive and visualize a phosphoproteomic-focused regulatory network. Phosphosites in kinases and phosphatases showing significant positive or negative correlations with the predominant YES1 phosphosite were subsequently classified.

**Figure 8:**
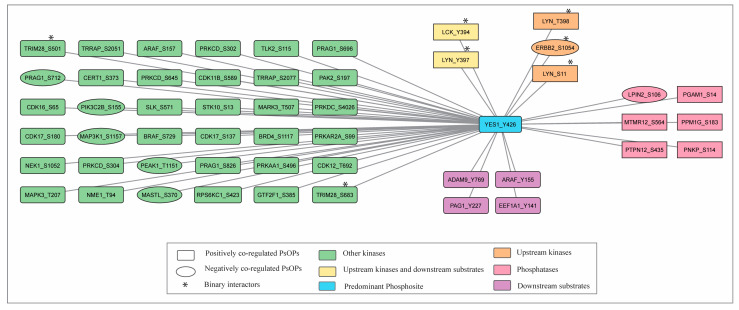
Network of co-modulated kinases, phosphatases, upstream regulators, and candidate downstream substrates of YES1 predominant site Y426.

From the positively co-modulated datasets, we identified 36 kinases, whereas the negatively co-modulated datasets yielded 6 kinases (Figure 8; Supplementary Table S8). The phosphosites on positively co-differentially modulated kinases, such as LYN (Y397), LCK (Y394), and BRAF (S729), are associated with inducing its enzymatic activity. The kinases, including MAPK3 (T207), PRKAA1 (S496), and GTF2F1 (S385), are positively co-differentially modulated with YES1 (Y426); however, the relevant phosphosites are linked to the inhibition of its enzymatic activity. Similarly, YES1 (Y426) is negatively co differentially modulated with PEAK1 (T1151), MASTL (S370), ERBB2 (S1054), PRAG1 (S712), PIK3C2B (S155), and MAP3K1 (S1157).

Further, we examined the potential upstream regulators among the co-differentially abundant kinases by comparing our data with data fetched from experimentally validated regulators, kinase prediction tools and Johnson et al. (2023) [41]. No experimentally validated upstream regulators are known for Y426 of YES1. Our analysis revealed LCK (Y394) and LYN (Y397, S11, and T398) as positively co-modulated upstream regulators and ERBB2 (S1054) as a negatively co-modulated upstream regulator, all of which can stimulate phosphorylation at YES1 Y426 (Figure 8) (Supplementary Table S9). LCK (Y394) is fetched from Johnson et al. (2023) [41], HTP as well as from kinase prediction tools such as ochava, iKiP-DB and Networkin databases. LYN at Y397, S11 and T398 are predictive upstream regulators extracted from the Networkin database.

LCK (Y394), ADAM9 (Y769), LYN (Y397), PAG1 (Y227), EEF1A1 (Y141), ARAF (Y155), EFNB2 (Y304), and CAVIN1 (Y308) were the candidate downstream substrates that show positive co-modulation with YES1 (Figure 8; Supplementary Table S10).

### 3.7. Phosphatases Co-Modulated with YES1 Predominant Site

Phosphatases were assessed relative to the predominant site of YES1. Our analysis found that YES1 (Y426) is positively co-differentially modulated with five phosphatases, namely, PGAM1 (S14), MTMR12 (S564), PPM1G (S183), PTPN12 (S435), and PNKP (S114), and negatively co-modulated with one phosphatase, LPIN2 (S106) (Figure 8). As reported in PhosphositePlus, phosphorylation at S114 of PNKP phosphatase was linked with inducing and inhibiting its enzymatic activity. YES1 (Y426) is negatively co-modulated with LPIN2 (S106), where the relevant phosphosites are not linked with enzymatic activity (Supplementary Table S11).

### 3.8. YES1-Associated Phosphorylation Events Reveal Oncogenic Signaling

**Figure 9:**
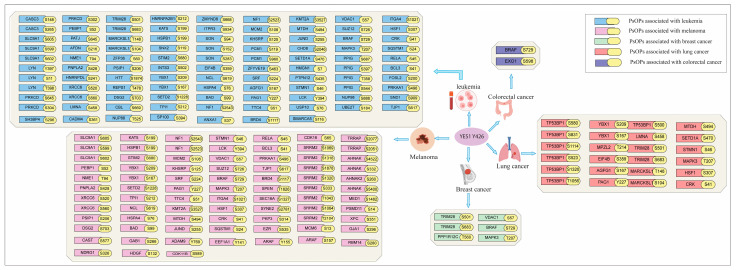
YES1 phosphosite Y426-associated positively co-modulated phosphoproteins overlap with cancer-specific biomarkers, showing site-specific phosphorylation events linked to breast cancer, leukemia, colorectal cancer, and lung adenocarcinoma.

Consistent with the reported oncogenic role of YES1 across multiple malignancies, including colorectal cancer, breast cancer, lung cancer, leukemia, and melanoma [5,16,69,70,71], we examined whether YES1-associated phosphoproteomic changes intersect with known cancer biomarkers. Biomarkers associated with these malignancies were retrieved from the DisGeNet database (Barcelona, Spain) and systematically compared with our positively co-modulated dataset, leading to the identification of common proteins presented in Figure 9. Subsequent site-specific analysis of these common proteins from our data revealed phosphorylation events linked to carcinogenic processes. In breast cancer, phosphorylation of YBX1 (S209) and CRK (S41) was observed, both of which have been implicated in tumorigenesis. In leukemia, phosphorylation of LYN (Y397), PRKCD (S645), and ZFP36 (S60) was identified, suggesting their involvement in leukemia-associated oncogenic signaling. Notably, phosphorylation sites including YBX1 (S209), BAD (S99), MCM2 (S108), LCK (Y394), ITGA4 (S1021), and CRK (S41) are known to promote carcinogenesis, supporting the biological relevance of our findings. At the protein level, BRAF and EXO1 are known to induce carcinogenesis and were identified in colorectal cancer [72,73], while in lung adenocarcinoma, VDAC1 and MAPK3 were associated with tumorigenesis [74,75]. Collectively, these results indicate that YES1-associated co-modulated proteins converge on cancer-type-specific oncogenic pathways through distinct phosphorylation events.

## 4. Discussion

YES1 is a non-receptor tyrosine kinase of the Src family kinases (SFKs) that regulates key cellular processes, including survival, growth, differentiation, apoptosis, cell–cell adhesion, and cytoskeletal remodeling, and plays a central role in controlling multiple cancer-associated signaling pathways [5]. YES1 is frequently amplified and overexpressed in numerous tumor types, including lung, breast, ovarian, colorectal, and skin cancers, where it promotes cell proliferation, survival, invasiveness, and metastatic progression [76]. Beyond cancer, it is also involved in Hepatitis E Virus (HEV) infection and inflammatory and fibrotic diseases [8]. Recent studies on YES1 kinase highlight its emerging significance as a therapeutic target and biomarker in various cancers, including lung, breast, and bladder cancers [6]. YES1 overexpression and activation are strongly linked with resistance to tyrosine kinase inhibitors (TKIs) and chemotherapeutic drugs such as EGFR and HER2 inhibitors. For example, YES1 amplification mediates acquired resistance to EGFR TKIs in non-small cell lung cancer, and its inhibition by dasatinib reverses resistance and reduces tumor growth in preclinical models [6]. Novel selective YES1 inhibitors, such as CH6953755 and NXP900, are being developed and demonstrate potent antitumor activity in YES1-amplified cancers [5].

While the present study provides valuable insights into predominant phosphosites in YES1 based on data generated from the bottom–up proteomic strategy, it is important to acknowledge the inherent complexity of the proteome and the limitations associated with this approach. Shotgun proteomics primarily captures canonical protein identities from peptide-level evidence and does not directly resolve intact protein species or the full spectrum of proteoforms arising from alternative splicing, post-translational modifications, sequence variants, or proteolytic processing. Consequently, distinct proteoforms with potentially divergent biological functions may not be fully captured or distinguished in the current analysis. Importantly, even within these methodological constraints, systematic analysis of large-scale phosphoproteomic datasets enables the identification of recurrent and functionally informative phosphorylation events in YES1.

In this context, our comprehensive study of global phosphoproteomic datasets systematically identified Y426 as a predominant phosphosite in YES1 kinase. This activation-loop phosphosite Y426 is important for YES1 catalytic function, as its phosphorylation is associated with induced kinase activity. Identification of multiple protein phosphosites that either positively or negatively co-modulate with YES1 phosphosite Y426 significantly broadens our understanding of its signaling context. These co-modulated phosphosites in other proteins are associated with essential biological processes such as cytoskeletal remodeling, cell cycle regulation, transcription, apoptosis, and carcinogenesis. This finding is consistent with YES1’s well-established multifunctional role in regulating cell growth, adhesion, migration, and survival pathways [5].

This study also explores the potential upstream regulators and candidate downstream substrates which are co-modulated with YES1 predominant phosphosite. LYN (S11/T398/Y397) and CBL (S669) were identified as the key binary interactors that are positively co-modulated with YES1 (Y426). LYN is an Src family kinase mainly expressed in immune cells, and phosphorylation at its activation-loop site Y397 is associated with increased kinase activity [77]. YES1 and LYN have been shown to phosphorylate EGFR at Y1101, a modification that promotes EGFR nuclear translocation [1]. However, a direct physical interaction between these kinases has not yet been experimentally demonstrated and requires further investigation. CBL (Casitas B-lineage lymphoma) is a family of E3 ubiquitin ligases that regulate signal transduction pathways by attaching ubiquitin to other proteins. Yes1’s binding to c-Cbl is phosphotyrosine-dependent, primarily through its SH2 domain that recognizes phosphorylated tyrosines on c-Cbl [78]. It phosphorylates key tyrosine residues in c-Cbl, such as Y700, Y731, and Y774, which are major phosphorylation targets in activated T cells and critically regulate immune cell functions, including protein-tyrosine kinase activation and adaptor protein recruitment during T cell receptor signaling [79]. While the functional significance of these well-characterized tyrosine phosphorylation sites is detailed, less is known about the specific regulatory roles of phosphorylation at S669 in c-Cbl. This suggests that S669 phosphorylation may be involved in specific, perhaps less common, regulatory mechanisms or signaling pathways.

In our analysis, LCK harboring a phosphosite Y394 and LYN phosphosite Y397 were identified as upstream regulators, candidate downstream substrates, and binary interactors that were positively co-modulated with the YES1 phosphosite Y426. Existing studies show that phosphorylation of LCK at Y394 occurs by autophosphorylation and stabilizes the activation loop of the kinase domain in an open, active conformation, which is essential for LCK’s full kinase activity [80]. This phosphorylation promotes T-cell receptor (TCR) signaling by enabling LCK to phosphorylate downstream targets, initiating T-cell activation. Conversely, dephosphorylation of Y394 negatively regulates LCK activity, thus serving as a key switch in controlling T-cell immune responses [81,82]. YES1 and LCK, two SRC family kinases, are both known to phosphorylate YAP1 at Y357, promoting its nuclear localization and transcriptional activation, thereby linking them as important regulators of the Hippo signaling pathway and downstream pro-proliferative and anti-apoptotic gene expression [83,84,85].

HER2 (ERBB2) with the phosphosite S1054 was found to be an upstream regulator as well as a binary interactor that is negatively co-modulated with YES1 phosphosite Y426. HER2 is already known to interact directly with YES1, and it is also a known upstream regulator of YES1. YES1’s binding and activation of HER2 play crucial roles in mediating resistance to HER2-targeted treatments, making YES1 a potential target for overcoming therapy resistance in HER2-driven cancers [22,76]. Existing studies indicate that the phosphorylation of HER2 at S1054 is associated with regulatory effects on HER2 signaling and can serve as a predictive biomarker for sensitivity to HER2 inhibitors such as lapatinib and neratinib in breast cancer, and increased phosphorylation at S1054 may represent a feedback response to anti-HER2 blockade in tumors, potentially linked with treatment resistance mechanisms [86].

Phosphosite Y426, located within the activation loop of the kinase domain, is conserved across Src-family members and is a well-established autophosphorylation site. In our data, LCK and HER2 (ERBB2) were shown to be co-modulated with this site and were predicted to be upstream regulators of YES1 (Y426). Interactions with these upstream regulators probably trigger YES1 conformational relief, disrupting intramolecular inhibition and promoting autophosphorylation rather than direct phosphorylation by the partners. Such mechanisms are well-supported by the structural biology of Src kinases [8,87].

Despite the expanding interest in YES1 as an oncogenic driver and therapeutic target, detailed phosphosite-focused information remains limited. Our phosphosite-focused, systems-level analysis addresses this gap by prioritizing Y426 as a frequently repeated, predominant, and highly co-modulated phosphorylation hotspot that links YES1 to extensive oncogenic signaling networks. The consistent co-modulation of Y426 with functionally important phosphosites across various biological processes and cancer-related pathways highlights its potential utility as a robust molecular marker. Importantly, prioritizing this high-confidence phosphosite offers a rational basis for target validation, supporting the discovery of phospho-specific biomarkers and therapeutic strategies for modulating YES1-driven signaling in cancer.

## 5. Conclusions

Our study presents a global overview of phosphoproteomic-focused modulation of YES1 across diverse biological contexts. By examining a large-scale human phosphoproteomic dataset, we identified Y426 as the predominant phosphosite of YES1, as it is the most frequently detected site across diverse experimental conditions. Our findings show that co-modulated phosphosites are involved in key biological processes such as transcriptional control, cell cycle regulation, cytoskeletal dynamics, apoptosis, and carcinogenesis. Comparison with DisGeNet cancer biomarkers revealed that protein phosphosites associated with YES1 overlap with established, site-specific cancer biomarkers across multiple malignancies. Collectively, our findings highlight the central importance of phosphosite-specific regulation in shaping YES1 signaling and demonstrate that such site-resolved insights provide a powerful framework for prioritized target validation for YES1-driven cancers in the future.

## 6. Limitations

This study summarizes phosphorylation events detected across YES1 that may arise from distinct proteoforms rather than occurring concurrently on a single protein species. Accordingly, while the present analysis offers a global view of YES1 phosphorylation patterns, it does not resolve proteoform-specific modification states. Future investigations employing top–down proteomics or targeted proteoform-resolved strategies will be necessary to more accurately delineate proteoform diversity. Although we applied stringent quality control filters such as *p*-value, localization probability ≥ 75%, A-score ≥ 13, and multi-layered filtering, our analysis of heterogeneous public phosphoproteomic datasets derived from diverse experimental platforms, conditions, and cell types introduces inherent variability. Complete normalization across these datasets remains challenging without access to raw data and uniform reprocessing pipelines. Therefore, these data-driven findings warrant additional validation in independent studies. The upstream regulators and the candidate downstream substrates of YES1 were identified through prediction-based strategies. Kinase prediction tools like NetworKIN or PhosphoSitePlus might loosely categorize these targets as “upstream kinases” based on correlation or pathway data. Hence, these upstream regulators and candidate substrates should undergo further validation based on the global phosphomotif enrichment and co-phospho-abundant regulation across various cellular and experimental conditions.

## Data Availability

All data can be found within the manuscript and its Supplementary Materials.

## Supplementary Materials

The following supporting information can be downloaded at: https://www.mdpi.com/article/10.3390/proteomes14020017/s1, Figure S1. Venn diagram illustrating the shared PsOPs across the top experimental conditions; Table S1: Co-occurance of YES1 phosphosites; Table S2: Human cellular phopshoproteomics profiling data showing YES1 identification; Table S3: Human cellular phosphohoproteomics data showing YES1 differential regulation; Table S4: List of phosphosites on other proteins that positively co-modulate with YES1 (Y426); Table S5: List of phosphosites on other proteins that negatively co-modulate with YES1 (Y426); Table S6: Biological process of co-modulated protein phosphosites with YES1 (Y426); Table S7: Binary interactors co-modulating with YES1 predominant phosphosite; Table S8: Kinases co-modulating with YES1 predominant phosphosite; Table S9: Upstream regulators co-modulating with YES1 predominant phosphosite; Table S10: Downstream substrates co-modulating with YES1 predominant phosphosite; Table S11: Phosphatases co-modulating with YES1 predominant phosphosite.
