# Supplementary material for: Computational Phosphosite-Specific Network Analysis of YES1 Y426 Reveals Cancer-Associated Phosphorylation Patterns
Source: Proteomes. 2026 Apr 16;14(2):17. doi: 10.3390/proteomes14020017 (PMC13108085; doi:10.3390/proteomes14020017)
Supplement: Supplementary file 1 [file proteomes-14-00017-s001.zip › Supplemetary Figure S1.pdf]

Computational Phosphosite-Specific Network Analysis of YES1 Y426 Reveals Cancer-Associated Phosphorylation Patterns

Afreen Khanum, Leona Dcunha, Suhail Subair, Athira Perunelly Gopalakrishnan, Akhina Palollathil[1,*], Rajesh Raju[1,*]

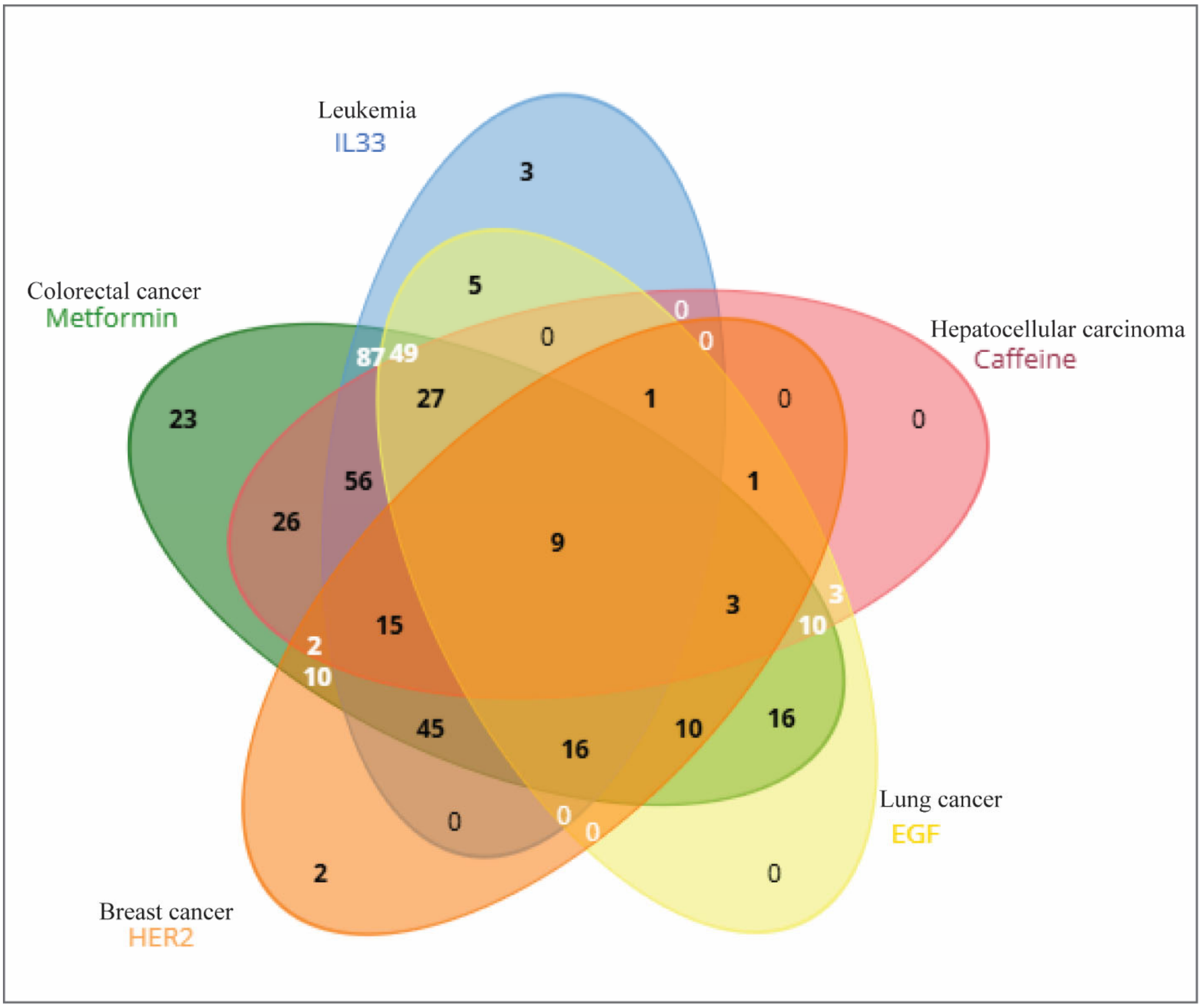

Supplementary Figure S1. Venn diagram illustrating the shared PsOPs across the top experimental conditions.
